# Spatiotemporal distribution of grassland NPP in Gansu province, China from 1982 to 2011 and its impact factors

**DOI:** 10.1371/journal.pone.0242609

**Published:** 2020-11-23

**Authors:** Meiling Zhang, Xiaoni Liu, Stephen Nazieh, Xingyu Wang, Teddy Nkrumah, Shanglang Hong

**Affiliations:** 1 Center for Quantitative Biology, College of Science, Gansu Agricultural University, Lanzhou, China; 2 Natural Resource Ecology Laboratory, Colorado State University, Fort Collins, CO, United States of America; 3 Department of Ecosystem Science and Sustainability, Colorado State University, Fort Collins, CO, United States of America; 4 College of Prataculture, Gansu Agricultural University, Lanzhou, China; Chinese Academy of Sciences, CHINA

## Abstract

The modified Carnegie–Ames–Stanford Approach (CASA) model based on the comprehensive and sequential classification system of grasslands (CSCS, a unique vegetation classification system) was used to determine grassland net primary production (NPP) in Gansu province from 1982 to 2011 and its spatio-temporal variability. The relationship between NPP and climate drivers was analyzed. The results showed that annual NPP of grasslands in Gansu province averaged 139.30 gC m^-2^ yr ^-1^ during the study period. NPP decreased from southeast to northwest across the province. Grassland NPP showed an increasing trend during the period 1982–2011, and the increase rate over the whole period was 92.91%. The highest NPP appeared in summer with more precipitation and higher cumulative temperature conditions; while the lowest values existed in winter. The largest correlation coefficient was found between the average annual NPP and the average annual precipitation (r = 0.77), followed by annual NPP and solar radiation (r = 0.70) or NDVI (r = 0.69), Annual NPP had no significant correlation with annual cumulative temperature (>0°C) or moisture index (K-value). Thus, precipitation is the major controlling factor on the average annual NPP in Gansu grassland. Solar radiation and NDVI also have important effects on grassland NPP in Gansu. These results may provide basic information for sustainable development and utilization of grassland and for the improvement and protection of the ecological environment as well.

## Introduction

Net Primary Productivity (NPP), which is equal to plant gross primary productivity (GPP) minus its autotrophic respiration (R_A_), is the energy source of primary consumers like livestock and wildlife [1]. NPP reflects the efficiency of plant fixation and transformation of photosynthetic products and is a key process of the carbon cycle. Grassland NPP, the result of interactions among soil, grass, and livestock in the grassland ecosystem and its external environmental factors, is an indicator of the productivity of grassland vegetation under natural conditions [2].

With the development of remote sensing technology, satellite data has become the most powerful means to assess NPP in terrestrial ecosystems [3, 4]. Estimating grassland NPP using mathematical models has become an important and widely accepted research method. Among these models, a light-use efficiency model Carnegie–Ames–Stanford Approach (CASA), which is based on remote sensing data and uses resource balance as theoretical basis, has been widely used to simulate the spatial distribution and variation of regional scale NPP [5, 6]. Donmez et al. [7] used the CASA model to estimate the current and future spatial distribution of NPP in a Mediterranean watershed, and obtained reasonable results. Huang et al. [8] improved the CASA model and used it to estimate the annual net primary productivity of the Colorado Plateau and found that the model is reliable. Zhang et al. [9] used the CASA model to estimate the vegetation NPP of alpine grassland on the Qinghai-Tibet Plateau in China, this laid a foundation for elucidating the characteristics and drivers of NPP of this plateau grassland ecosystem. The CASA model also achieved reliable results in the NPP estimation study in Inner Mongolia grasslands in China [10, 11] and Mongolia [12].

The Comprehensive and Sequential Classification System of grasslands(CSCS), a unique vegetation classification system (mainly for grassland), is dependent on quantitative measurement indices such as >0°C annual cumulative temperature (Σθ) and moisture index [13]. The CSCS recognizes 10 broad vegetation categories: tundra alpine steppe (TAS), frigid desert (FD), warm desert (WD), semi-desert (SD), steppe (ST), savannah(SA), temperate humid grassland (THG), temperate forest (TEF), subtropical forest (STF), and tropical forest [13, 14] Among them, 6 categories are present in Gansu province, China, including TAS, FD, SD, ST, THG, and TEF [13]. Based on the CSCS, the CASA model was modified and was used to reconstruct the spatio-temporal changes of NPP in China’s grassland ecosystem from 2004 to 2008 [15]. Gansu province is located at the intersection of the three plateaus of Loess, Qinghai-Tibet and Mongolia in northwest China, and has a semi-arid to arid continental climate. The landscape in Gansu is very complex, including mountains, basins, deserts, and gobi. Due to severe environmental and anthropogenic destruction, there appear to be some ecological problems such as grievous desertification, soil erosion and salinization in this region [16]. In the study, meteorological data and satellite remote sensing data were used to simulate the grassland NPP in Gansu, China, from 1980 to 2011 by the modified CASA model based on the CSCS. The objective of the present study is to provide basic data for sustainable development, utilization, improvement and protection of grassland ecological environments in arid and semi-arid regions.

## Materials and methods

### Research area

Gansu province covers an area of approximately 42.58 million hectares and is located between the Tibetan and Loess plateaus (32.58°-42.78° N, 92.35°-108.71° E) [5]. Most areas of Gansu province are dominated by semi-arid and arid climates and belong to the temperate monsoon climate. The annual average temperature ranges from 0°C in the northwest to 16°C in the southeast. The annual average precipitation increases from 36.6 mm in the northwest to 734.9 mm in the southeast. Most precipitation is concentrated as intensive storms from June to September. The annual average evaporation is much higher than the amount of precipitation. Annual total solar radiation is about 4600~4800 MJ m^-2^, and gradually decreases from the northwest to the southeast. Elevation ranges from 1500 to 3000 m above sea level.

The grassland area in Gansu is 15.75 million hectares, accounting for 36.99% of the total land area. Of this, the 15.65 million hectares of natural grassland, accounting for 99.34% of the total grassland area, is one of the major animal husbandry bases in China [13]. The distribution of grassland shows obvious vertical zone. Warm arid, warm temperate semi-desert, cool temperate, dry temperate semi-desert, and cold temperature and moist temperate coniferous forest are the most important grassland types in Gansu province [13].

### The modified CASA model

The CASA model is a light-use efficiency model, driven by remote sensing data, meteorological data, vegetation types and soil types, Eq (1):

$$\mathrm{NPP}(x,t)=\mathrm{FAPAR}(x,t)\times \mathrm{PAR}(x,t)\times \varepsilon_{\max}(x,t)\times T_{1}(x,t)\times T_{2}(x,t)\times W_{\varepsilon }(x,t)$$ (1)

NPP(*x*, *t*) is the vegetation NPP in the geographic coordinate system of a given location *x* and time *t*. FAPAR(*x*, *t*) is the fraction of absorbed photosynthetically active radiation, PAR(*x*, *t*) represents the incident photosynthetically active radiation, ε_max_(*x*, *t*) is the maximum light-use efficiency variable, T_1_(*x*, *t*) and T_2_(*x*, *t*) are the temperature stress coefficients, and W_ε_ (*x*, *t*) represents the moisture stress coefficient.

The calculation of FAPAR(*x*, *t*)、PAR(*x*, *t*)、T_1_(*x*, *t*) and T_2_(*x*, *t*) are the same as for the CASA model [17]. Here, two modifications were proposed for the CASA model based on the CSCS so that the evalution of grassland NPP and the classes/super-classes in the CSCS may achieve optimum coupling.

First, the maximum light use efficiency (ε_max_) was set to the same value for all vegetation types in the CASA model [17]. Here the ε_max_ for different grassland types was estimated by minimizing error between the observed NPP and estimated NPP at observed sites (Table 1). The principle of the minimum error can be expressed by Eq (2):

$$E(x)=\sum_{i=1}^{j}t_{i}^{2}x^{2}-2\sum_{i=1}^{j}s_{i}t_{i}x+\sum_{i=1}^{j}s_{i}^{2}\;x\in [u,v]$$ (2)

Where, *i* and *j* are the sample number of the vegetation, respectively. Here, *s*_*i*_ represents the observed NPP, while *t*_*i*_ represents the product of temperature, absorbed photosynthetically active radiation and water deficit stress. *x* is ε_max_ modeled for the vegetation. Meanwhile, *u* and *v* represent the lower and upper light use efficiency of the grassland types, respectively.

**Table 1:** The simulation value of the maximum light utilization rate(*ε*_max_) of Gansu.

Vegetation type	Observation site	The average of observation NPP (g C/m^2^/y r)	The range of observation NPP (g C/m^2^/y r)	*ε*_max_ max	*ε*_max_ min	*ε*_max_ estimated
TAS	HZ	490.7	58.39–708.43	0.74	0.04	0.16
FD	DH, AX	408.96	93.01–546.33	2.58	0.06	0.73
SD	GT, MQ, JT	396.79	24.8–602.97	2.4	0.01	0.61
ST	SD, GL, JY	398.22	107.32–370.99	0.84	0.02	0.26
THG	SD	489.64	70.44–1451.22	0.94	0.01	0.33
TEF	HJL,MAQ	575.83	28.4–1334.11	0.53	0.001	0.17

Note: Vegetation types: TAS: tundra alpine steppe, FD: frigid desert, SD: semi-desert, ST: steppe, THG: temperate humid grassland, and TEF: temperate forest

Observation sites: HZ: Hezuo, DH: Dunhuang, AX: Anxi, GT: Gaotai, MQ: Mingqin, JT: Jingtai, SD: Shandan, GL: Gaolan, JY: Jinyuan, HJL: Huajialing, and MAQ: Maqu

Second, moisture stress coefficients W_ε_ (*x*, *t*), were calculated using a soil moisture model that requires a variety of soil parameters which are difficult to obtain as credible values in the CASA model. To exclude several soil parameters, the classification index, >0°C annual cumulative temperature (Σθ) and moisture index (K) in CSCS were used and referred to the existing regional evapotranspiration model to estimate W_ε_ (*x*, *t*) using the following formula:

$$W_{\varepsilon }\left(x,t\right)=0.5+0.5\times \frac{\left(0.29K^{\frac{1}{2}}+0.6\right)\left(K\cdot L(K)+0.469K^{\frac{3}{2}}+9.33{\left(\sum \theta \right)}^{-1}\right)}{\left(K+0.469K^{\frac{1}{2}}+0.966\right)\left(L(K)+0.933K^{-1}\right)}$$ (3)

$$\mathrm{Where},\;L(K)=K+0.906K^{-\frac{1}{2}}+0.22$$ (4)

$$K=\frac{P}{0.1\sum \theta }$$ (5)

where K is moisture index; P is annual precipitation and Σθ is >0°C annual cumulative. Here, moisture index referred as K is expressed as the ratio between annual precipitation and >0°C annual cumulative temperature.

### NDVI and climate data

The remote sensing data of Advanced Very High Resolution Radiometer (AVHRR) NDVI from 1982–2000 was download from “ltdr.nascom.nasa.gov”. MODIS NDVI from 2001–2011 was download from the NASA Medium Resolution Imaging Radiometer data (https://ntrs.nasa.gov/search.jsp). The data were processed by radiometric correction using the NOAA standard method [18], atmospheric correction using the method of ozone absorption [19], Rayleigh scattering [20], stitching and cropping. Then, after through data fusion and time series reconstruction, the NDVI data set of the study area from 1982 to 2011 was obtained. The spatial resolution was 1 × 1 km, and the temporal resolution was 16 days. The maximum value composite (MVC) procedure was used to merge NDVI values from 16 consecutive days to the monthly NDVI data sets.

**Fig 1:**
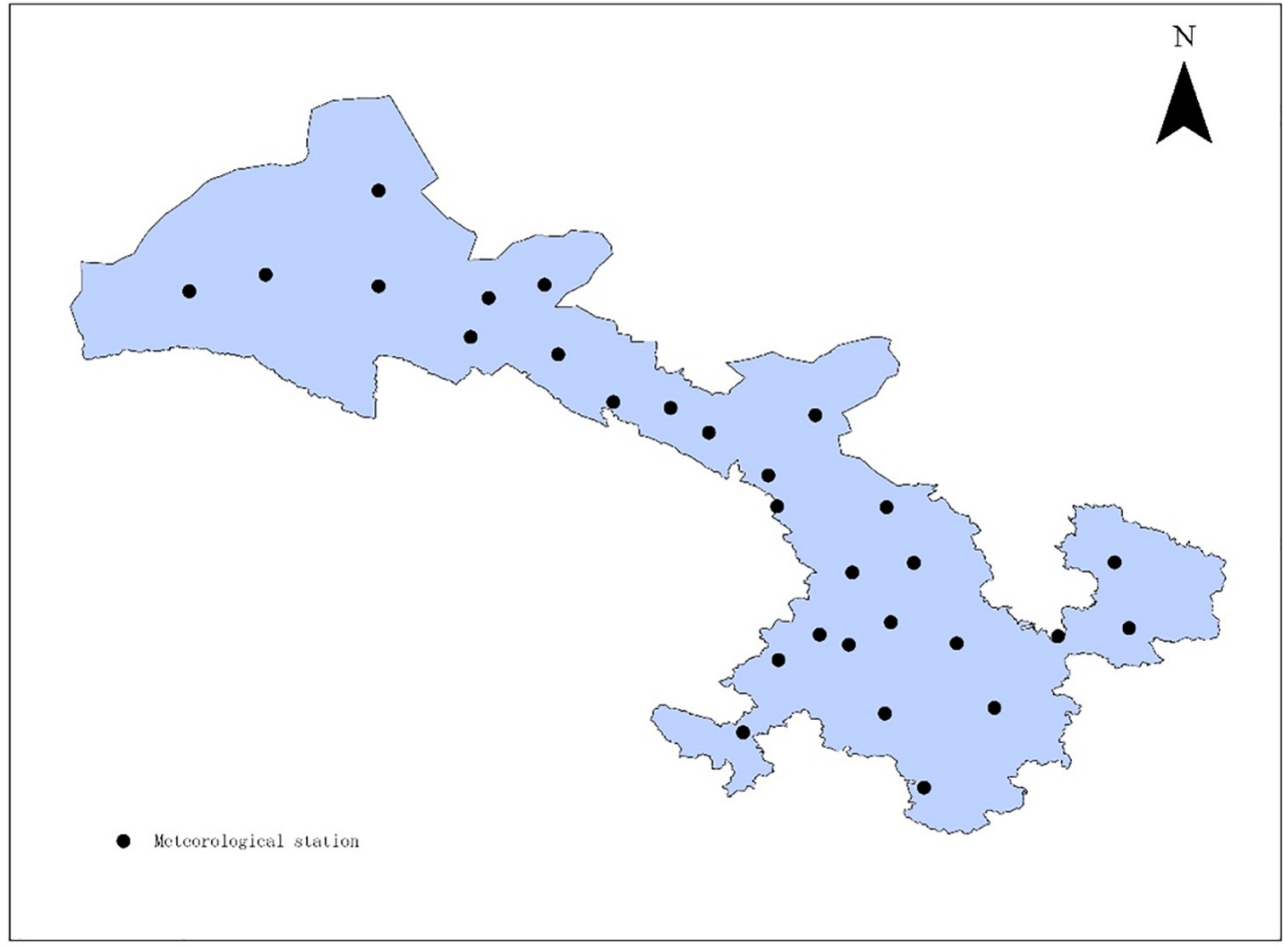
Spatial distribution of meteorological observation station in Gansu, China.

The meteorological data was acquired from the China Meteorological Science Data Sharing Service Network (http://data.cma.cn), including the monthly average temperature (°C) and monthly average precipitation (mm) of 29 meteorological stations in Gansu province (Fig 1) from 1982 to 2011, and the monthly total solar radiation (MJ m^-2^) of 15 stations around Gansu province. The Ordinary Kriging method in ARCGIS 9.1 software (ESRI, California, USA) was used for the interpolation of these parameters.

### Observed data

**Fig 2:**
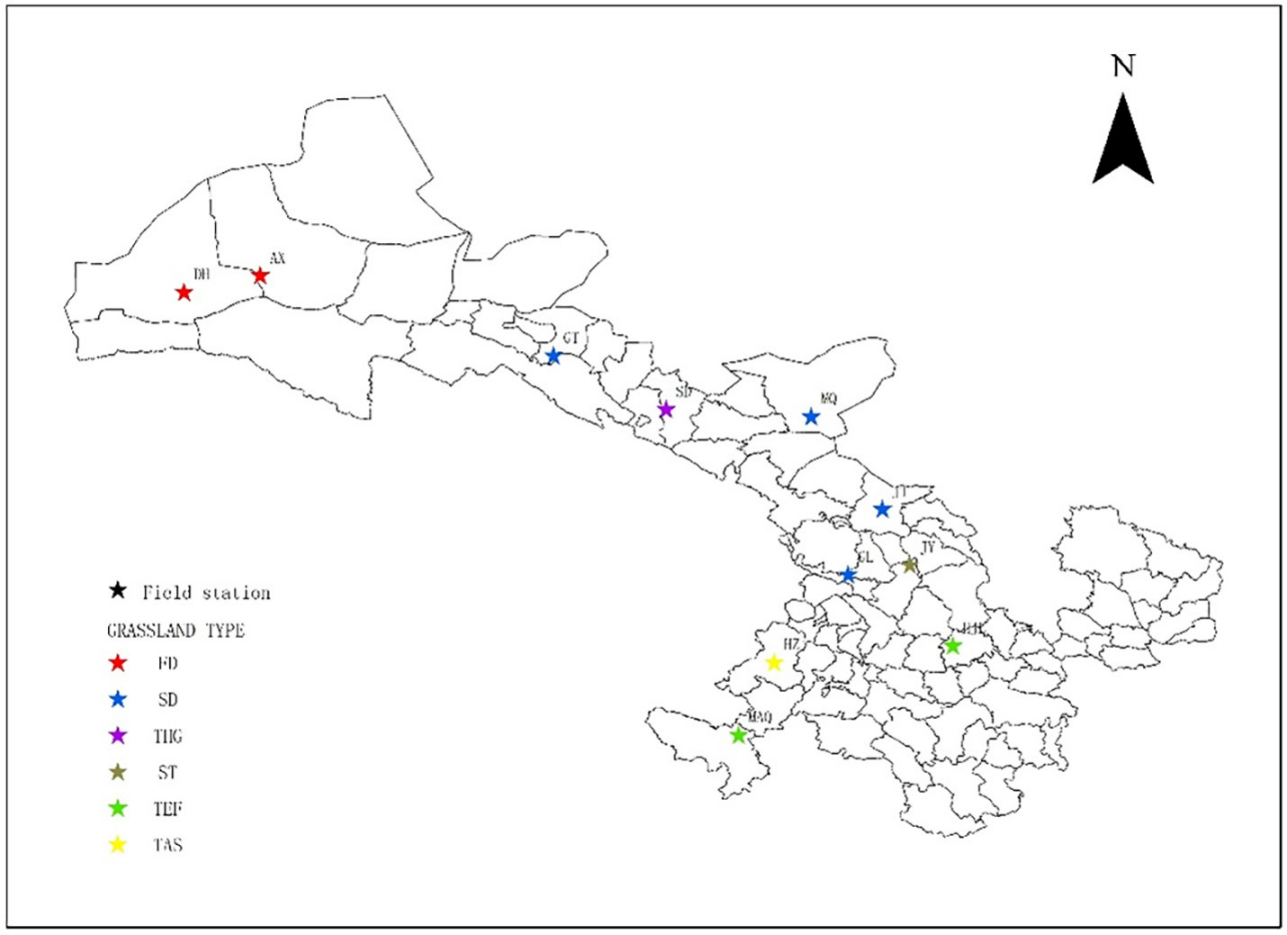
Spatial distribution of field observation station in Gansu, China. (See Table 1 for site abbreviations).

To validate the CASA model, the observed vegetation data were gathered from the data of the national grassland resource survey from 2005 to 2006 in China [15, 21], which were obtained from 11 grassland sites across Gansu province (Fig 2). The latitude, longitude, elevation, leaf area index, total biomass, and total NPP were documented for each of the grassland site. The vegetation types included tundra alpine steppe (TAS), frigid desert (FD), semi-desert (SD), steppe (ST), temperate humid grassland (THG), and temperate forest (TEF). The grassland NPP was the sum of aboveground and belowground NPP following our previous methods [15]:

$$NPP=B_{g}\times S_{b_{n}}\times \left(1+S_{u_{g}}\right)$$ (6)

Where, *B_g_* is dry biomass in units (g m^-2^); $S_{b_{n}}$ is coefficient converted to C units (g C m^-2^ yr^-1^) by a mass fraction of 0.475; $S_{u_{g}}$ is the ratio of aboveground and belowground biomass.

### Model validation test

**Fig 3:**
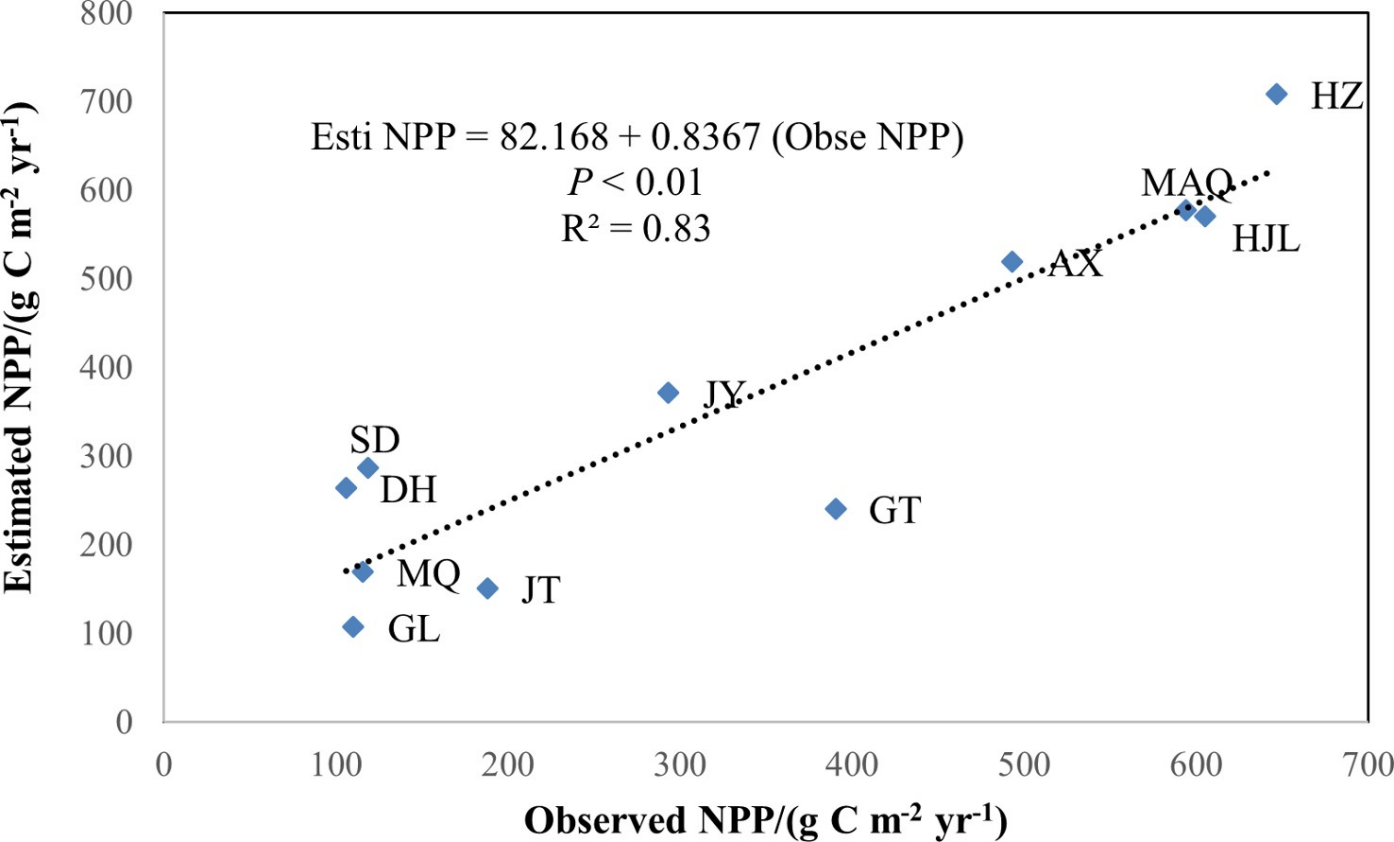
Comparison of estimated and observed NPP in Gansu, China from 1982 to 2011.

After the model modification, the observed data of 6 vegetation types of 11 observedsites in Gansu (Fig 2) from 2005 to 2006 were used to validate the modified model. The simple linear regression model was used to test the relationship between the estimated and the observed NPP for 11 sites (Fig 3). Coefficient of determination (R^2^) was calculated to show the strength of the relations.

### Correlation analyses

Using Eq (5), the correlation analyses were performed between the annual average NPP values of grassland in Gansu, China from 1982 to 2011, and annual average precipitation, >0°C annual accumulated temperature (Σθ), K value, solar radiation or NDVI. The correlation coefficient was tested by t test between grassland NPP and the above factors. The Durbin-Watson values were from 1.78 to 2.22, indicating that there were no time autocorrelation problems in the data used in the research.

$$R_{xy}=\frac{\sum_{i=1}^{n}\left(x_{i}-\bar{x}\right)\left(y_{i}-\bar{y}\right)}{\sqrt{\sum_{i=1}^{n}\left(x_{i}-\bar{x}\right)\cdot \sum_{i=1}^{n}{\left(y_{i}-\bar{y}\right)}^{2}}}$$ (7)

Where x_i_ represents the interpolated data; y_i_ indicates the measured data; x is the average value of the interpolated data; y is the average value of the measured data, and n represents the number of the sample.

## Results

### Validation of the modified model

It can be seen from Fig 3 that there is no overall overestimation or underestimation of the simulated values relative to the measured values. The correlation between the simulated NPP and the measured NPP of the Gansu grassland reached a significant level (R^2^ = 0.83, *P* < 0.01). It can be seen that the improved CASA model simulation results are reliable and suitable for the estimation of grassland NPP in Gansu, China.

### Spatial variation of annual average NPP

**Fig 4:**
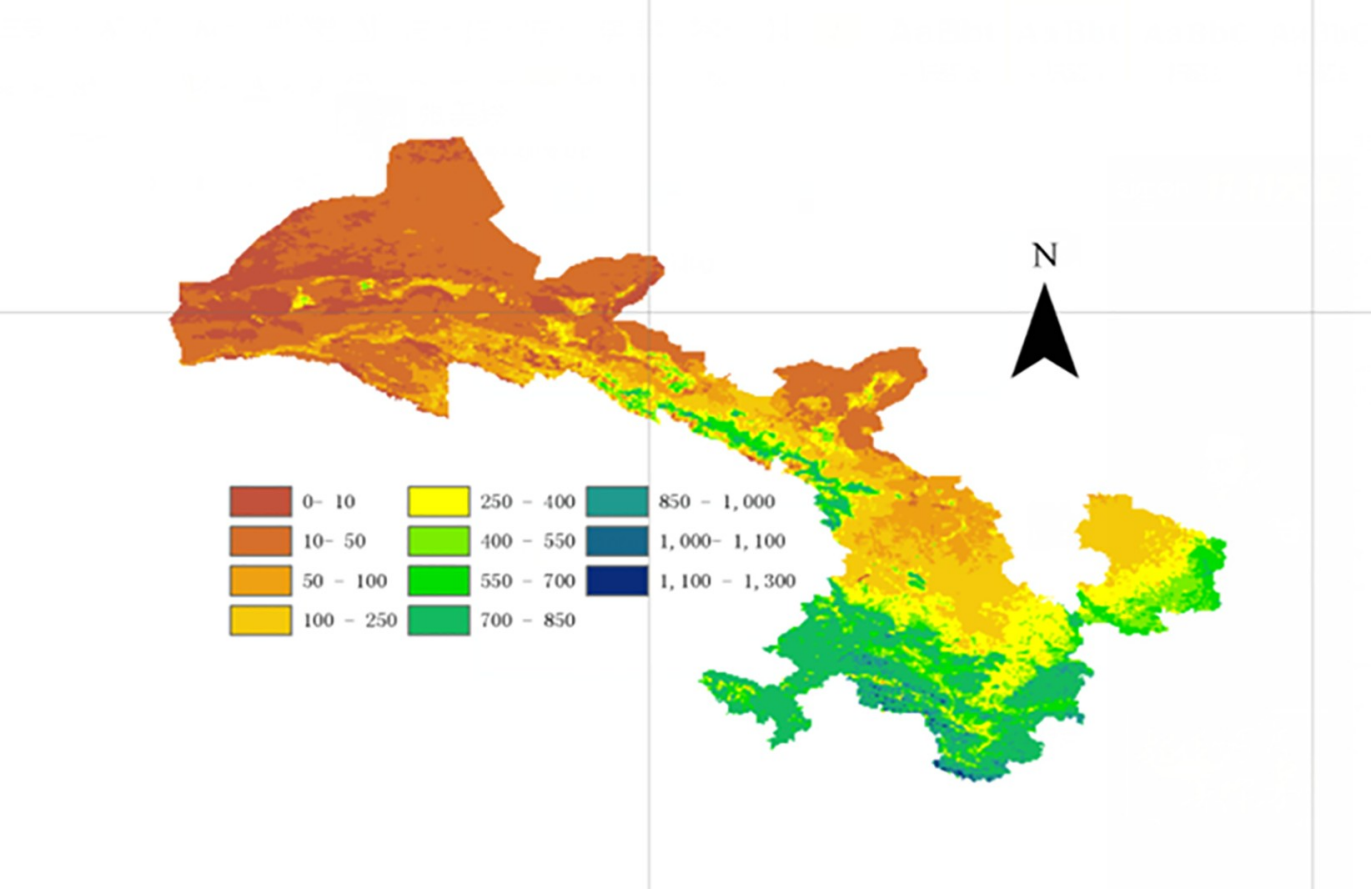
Spatial distribution of grassland NPP in Gansu, China from 1982 to 2011.

Fig 4 is the spatial distribution of the annual average NPP of Gansu grassland from 1982 to 2011. The annual average NPP of grassland in Gansu from 1982 to 2011 was 139.30 gC m^-2^ yr^-1^, with a gradual reduction from southeast to northwest. The high NPP value areas were concentrated in the Gannan Plateau and Longnan Mountain, while the low NPP value areas were distributed in the northwestern mountains. The largest NPP values were distributed, between 36.5°- 40.2°N and 98°-104°E. The grassland types in the Gannan Plateau include alpine meadows, mountain meadows and marshes. Due to the influence of the southwest monsoon climate from the Bay of Bengal, Gannan generally has abundant precipitation, sufficient solar radiation and fertile soil. The grassland annual average NPP of this area was between 279 and 512 gC m^-2^ yr^-1^. Longnan is the only region in the Gansu province that belongs to the Yangtze River system and has a subtropical climate. The grassland types are mainly mountain meadow, warm shrub and warm grass, which are dominated by subtropical humid climate, warm temperate humid climate, temperate semi-humid climate and alpine humid climate. Its annual average NPP was 146–409 gC m^-2^ yr^-1^ (Fig 4).

Longzhong, located in the central and eastern part of the Gansu province, is a temperate, semi-arid and semi-humid area and an important transition zone between China’s arid and humid climate. The average annual grassland NPP values were between 0 and 400 gC m^-2^ yr^-1^ from northwest to southeast, with an obvious latitude difference. The Qilian Mountains are located in the mid-latitude north temperate zone and belong to the temperate continental climate and the plateau mountain climate. The eastern part of Qilian Mountains is mainly alpine meadows and mountain meadows, and its annual average grassland NPP was between 300 and 400 gC m^-2^ yr^-1^. However, its western part is mainly alpine desert, alpine grassland and lowland meadow grassland, and its annual average NPP was 0 to 200 gC m^-2^ yr^-1^. The Hexi Corridor passes through arid and semi-arid areas, and contains warm grassland, lowland meadow, warm desert, warm grassland desert, and warm desert grassland, and the annual average NPP was from 0–400 gC m^-2^ yr^-1^ (Fig 4).

In the North Mountains and the western Hexi Corridor, the annual average grassland NPP was only 0–50 gC m^-2^ yr^-1^ (Fig 4). The climate is extremely dry, and its evaporation is always much higher than its precipitation. Its vegetation coverage is extremely low, and its dominant grassland types consist of warm deserts and lowland meadows.

### Temporal variation of annual average NPP

**Fig 5:**
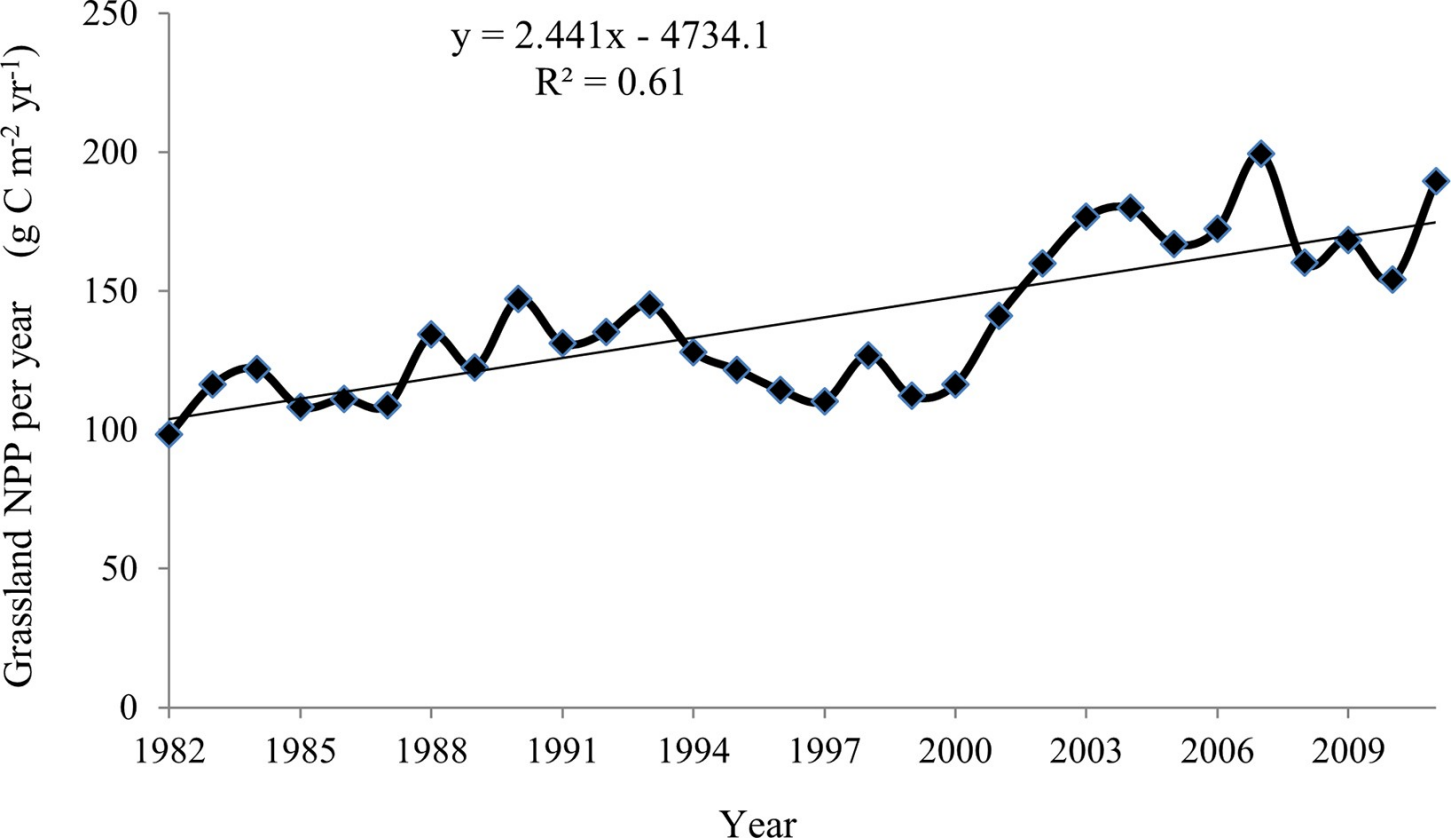
Interannual changes of grassland NPP in Gansu, China from 1982 to 2011.

The annual average NPP of grassland in Gansu from 1982 to 2011 varied between 98.26 and 199.40 gC m^2^ yr^-1^ (Fig 5). During the study period, although the annual average NPP fluctuated, it showed an upward trend. The minimum annual average grassland NPP value appeared in 1982 (98.26 gC m^2^ yr^-1^). The maximum values were obtained in 2011 and 2007, which were 189.55 and 199.40 gC m^2^ yr^-1^ respectively. Compared to the annual average grassland NPP in 1982, the value in 2011 increased by 92.91% (Fig 5).

**Fig 6:**
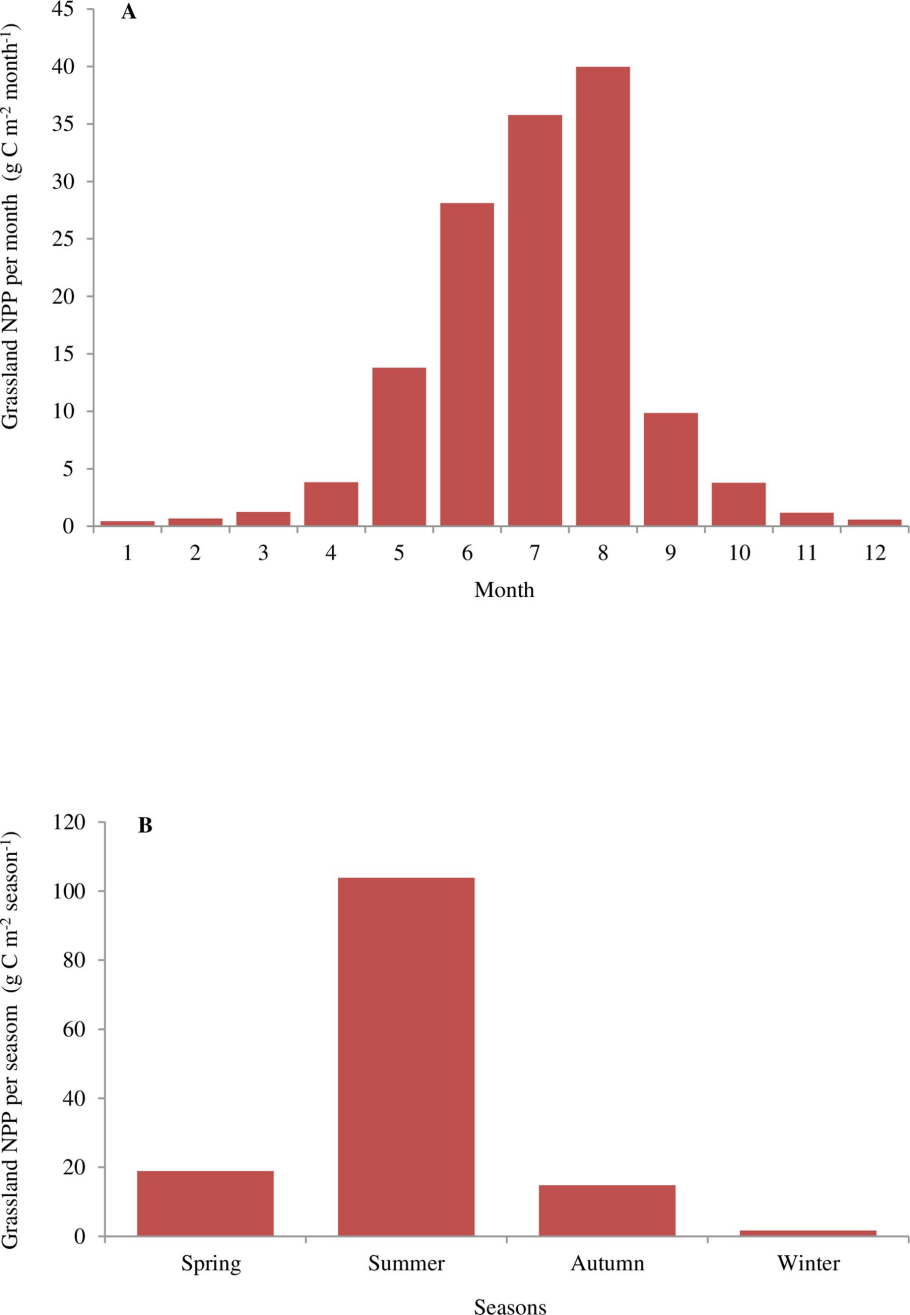
Monthly and seasonal variation of grassland NPP in Gansu, China from 1982 to 2011.

The monthly average NPP of the Gansu grassland from 1982 to 2011 was further analyzed (Fig 6A). The NPP values varied greatly from month to month due to the combined effects of precipitation and temperature. The accumulation period of grassland NPP mainly occurred from April to October, with good hydrothermal conditions. The total grassland NPP in the 7 months (from April to October) during the study period was 135.16 gC m^-2^, which was 97.03% of the total annual NPP. From November to March of the following year, the grassland NPP was relatively low (about 4% of the total annual; Fig 6A), due to low temperatures and slow growth of plants. It also can be seen from Fig 6A that the maximum monthly average NPP appeared in July and August, was 35.79 and 39.98 gC m^-2^ month^-1^, respectively; the minimum value appeared in January, which was 0.45 gC m^-2^ Month^-1^.

Fig 6B also shows the obvious seasonal differences in grassland NPP in Gansu, China during the study period. The largest grassland NPP was in summer (103.89 gC m^-2^, which was 74.58% of the total annual) because of sufficient solar radiation and good water and thermal conditions; However the smallest grassland NPP was in winter, being only 1.72 gC m^-2^ (1.23% of the total annual). Thus, hydrothermal conditions may be one of the decisive factors determining the grassland NPP in Gansu, China.

### The correlation between annual average NPP and climatic factors

**Fig 7:**
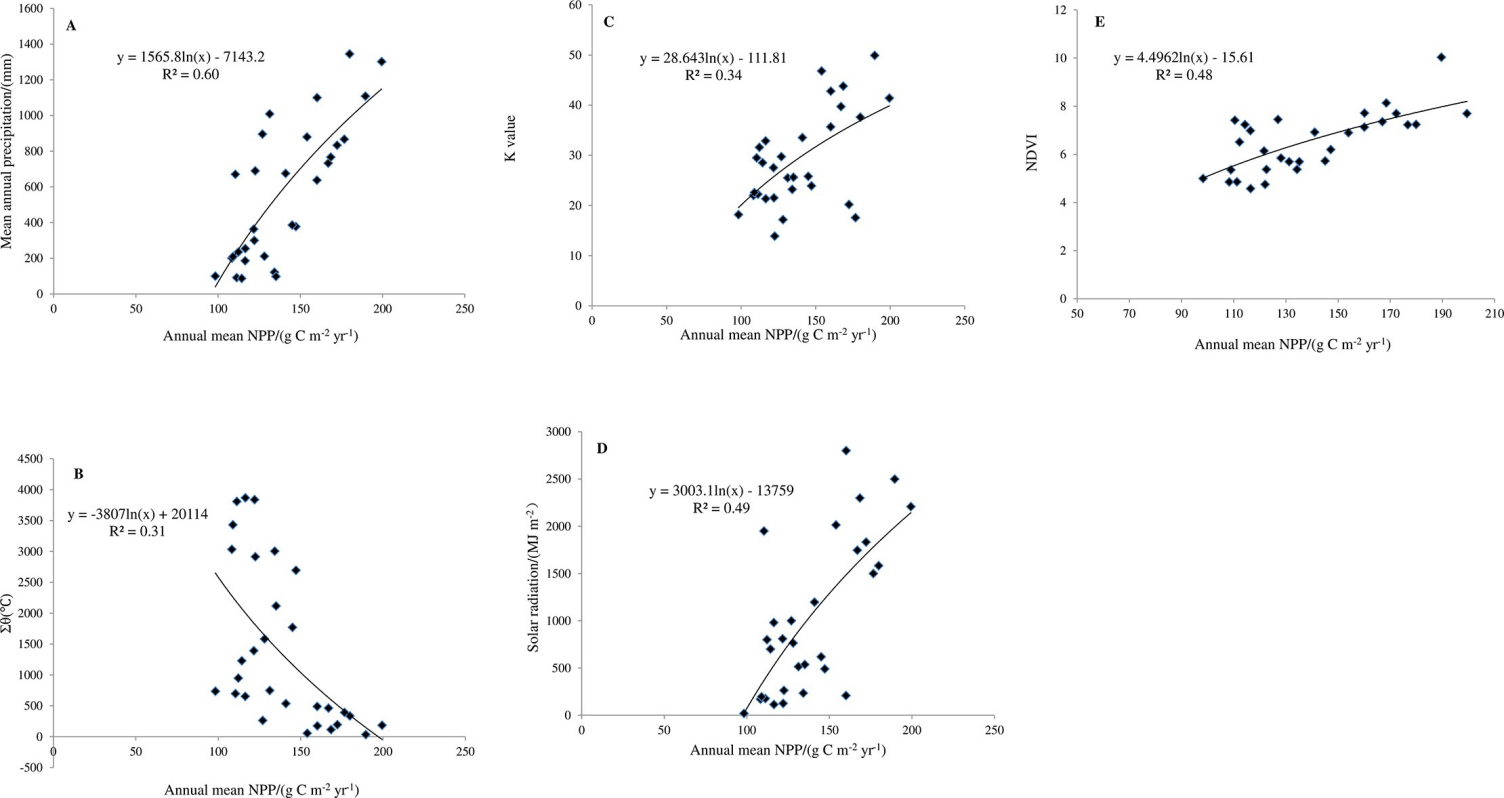
Correlation analysis between annual mean grassland NPP and climate factors in Gansu, China from 1982 to 2011.

The annual average grassland NPP in Gansu was significantly and positively correlated with annual average precipitation (*P*<0.01), and the correlation coefficient was 0.77 (Table 2 and Fig 7A). The annual average grassland NPP increased from 1982 to 2011 with the increase of precipitation, indicating that precipitation is one of the most important climatic factors influencing grassland vegetation productivity. The annual average grassland NPP was negatively correlated with >0°C annual accumulated temperature (Σθ), but it was not significant (*P*>0.05, R^2^ = 0.31; Table 2 and Fig 7B).

**Table 2:** Correlation coefficients between the annual average of grassland NPP and its related factors in Gansu during 1982~2011.

	Mean annual precipitation	>0 ^o^C annual cumulative temperature (Σθ)	K value	Solar radiation	NDVI
NPP	0.77**	-0.56	0.58	0.70*	0.69*

* and ** represent correlation is significant at the 0.05 and 0.01 level (2-tailed), respectively.

The annual average grassland NPP was positively correlated with K value, but it was also not significant (*P*>0.05, R^2^ = 0.34; Table 2 and Fig 7C). The annual average NPP was positively and significantly correlated with solar radiation and NDVI in the Gansu grassland (*P*<0.05), and the correlation coefficients were 0.70 and 0.69, respectively (Table 2 and Fig 7D and 7E). Solar radiation and NDVI exhibited a strong consistent trend of change with grassland NPP, indicating that both of them might play an important role in determing the grassland NPP. Therefore, solar radiation and NDVI had significant positive effects on grassland NPP, while K value had little effect.

## Discussion

Using the modified CASA model, the annual average NPP of grassland vegetation in the Gansu Province, China from 1982 to 2011 was simulated, and its spatio-temporal distribution pattern and relationship with various related factors were analyzed. There were significant differences in spatial distribution of grassland NPP during the study period. The annual average NPP gradually decreased from southeast to northwest: Gannan Plateau, Longnan Mountain and Qilian Mountain were the highest-value areas of grassland NPP in Gansu province, while the western part of the Hexi Corridor and North Mountain had the lowest values. Using the CASA model, Wei et al. [22] simulated the NPP of the Gansu grassland ecosystem in 2005, and obtained an average annual NPP of 139.15 gC m^-2^ yr^-1^. In this study the annual average grassland NPP was 166.95 in 2005, and 139.30 gC m^-2^ yr^-1^ from 1982 and 2011, respectively. The spatial distribution characteristics were also manifested as gradually decreasing from southwest to northeast [22]. Similarly, the annual average NPP of the Gansu vegetation from 2000 to 2010 also showed significant regional differences, with a higher annual average NPP in the southeast and a lower annual average NPP in the northwest [16]. In Wang et al. [23]’s study, the annual average NPP of the Gannan grassland from 2001 to 2008 was found to be 483.41 gC m^-2^ yr^-1^, falling within the results of this study, which was from 279 to 512 gC m^-2^ yr^-1^ between 1982 and 2011. Although our results displayed a good consistency with previous results, we could not exclude the possibility that the modified CASA model may have underestimated the grassland NPP in Gansu province, giving the ignore of the NPP of oases in the model evaluation. There exist a number of systematic biases causing grassland NPP to be underestimated or overestimate by model simulation, such as the accuracy and reliability of data used in the model [15], the uncertainty and sensitivity of parameters in inversion [14] and the ignore of specific vegetation types. Therefore, the simulated grassland NPP by model might underestimate the true NPP, but the extent of this underestimate is poorly known.

From the temporal pattern, the grassland NPP of Gansu exhibited an obvious upward trend from 1982 to 2011. The average annual NPP of vegetation in Gansu from 2000 to 2010 also showed an increasing trend [16]. From 1961 to 2013, China’s grassland carbon storage showed a significant increasing trend, with an average annual increment of 9.62 Tg C yr^-1^ [24]. The annual average grassland NPP in China also showed an increasing trend from 2004 to 2008, with an average annual increase rate of 5.02% [15]. Many studies around the world have found that NPP in various grassland types has increased in recent decades [25, 26]. Recently, using the MODIS-NPP dataset, Zhao et al. [27] found that the grassland NPP in Inner Mongolia from 2000 to 2014 had an annual rate of increase of 4.53 g C m^-1^ yr^-1^. According to the characteristics of monthly and seasonal variation, the maximum grassland NPP in Gansu was obtained in the summer when there were good precipitation and temperature conditions, while plants almost stopped growing in winter, and the grassland NPP value is the smallest. This is consistent with the findings of Wei et al. [22]and Zhang et al [14]. However, in the grassland NPP study of Uruguay from 2000 to 2010, it was found that the highest production occurred in spring and decreased sharply in early summer [28]. This may be because Uruguay belongs to the subtropical monsoon humid climate, with rainy, mild and humid weather conditions throughout the year, and the seasons change gradually.

Correlation analyses show that the grassland NPP in Gansu from 1982 to 2011 was significantly and positively correlated with the annual average precipitation. The precipitation in the Gansu province decreases from southeast to northwest, and the climate transitions from humid to semi-humid and semi-arid to arid. The spatial variation of precipitation is in agreement with the spatial pattern of grassland NPP, suggesting the importance of precipitation for Gansu grassland productivity. Precipitation also was reported to be the major climatic factor driving the increase of soil carbon in China’s grassland from 1961 to 2013 [24]. Similarly, the precipitation in Inner Mongolia from 1982 to 2003 has a strong correlation with its grassland NPP, while its temperature was weakly correlated with its NPP [29]. However, the study by Liu et al. [16] showed that the correlation of annual average NPP in the Gansu grassland with temperature was the highest from 2000 to 2010, followed by that with precipitation. In the study of grassland in southern China, it was also found that temperature, rather than precipitation, was the main limiting factor for grassland productivity [30]. Thus, the effects of precipitation and temperature on the productivity of vegetation may vary greatly depending on the study location. Piao et al. [31] found that the increase in NPP in the central and southeastern grasslands of the Qinghai-Tibet Plateau was mainly due to the increase of precipitation. Subsequent studies also showed that the annual precipitation was the major driver of productivty in the Qinghai-Tibet Plateau [32]. Using the improved CASA model to simulate the spatial distribution of grassland NPP in the Gannan pastoral area, Yang et al. [33] found that grassland NPP was closely related to the variation in hydrothermal conditions and the change in wetness would be the major driver of NPP change in the pasture of the Gannan Plateau. The study of grassland NPP in Inner Mongolia using the CASA model showed that the relationship between the NPP in meadow grasslands and precipitation and temperature both were very high, and the correlation of NPP with temperature was higher [10], while, the productivity of typical grassland and desert steppe were mainly controlled by precipitation [10]. A significant positive correlation between precipitation and the above ground NPP of the meadow, and desert steppes on the Inner Mongolian Plateau between 2011 and 2013 has been reported [34]. Donmez et al. [7] found that the vegetation NPP in the Mediterranean during growing season was strongly correlated with solar radiation and precipitation, and precipitation was an important controller of productivity variation. It can be concluded that rainfall may be the most decisive factor for grassland vegetation productivity in the arid and semi-arid areas. However, rainfall may not be as important for grassland productivity in rainy areas.

In this study, the annual average grassland NPP from 1982 to 2011 was also significantly and positively correlated with solar radiation or NDVI. In the Sanjiangyuan area, the correlation coefficient between vegetation NPP and NDVI was the highest, followed by that between vegetation NPP and temperature, and the lowest coefficient was that between vegetation NPP and precipitation or solar radiation [15]. According to Wei et al. [19], except for the slopes greater than 30°, the grassland NPP in Gansu in 2005 increased with increasing slopes, indicating that the productivity of the Gansu grassland increased with the increase of solar radiation. NDVI reflects the density of plant coverage in a region which is naturally associated with local solar radiation. In general, high precipitation and solar radiation may be beneficial for plant growth and vegetation coverage, and consequently maintain high NDVI. Thus, the effects of NDVI, solar radiation and precipitation on grassland NPP are not independent of each other. Each factor interferes with the others, eventually forming a complex impact mechanism. In order to further reveal the influence mechanism of various factors on grassland productivity, some larger scale simulation studies, more accurate productivity models and more grassland measured data are all needed in the future.

In summary, the modified CASA model was used to simulate grassland NPP in Gansu, China from 1982 to 2011, and its spatio-temporal pattern was analyzed. It gradually decreased from southeast to the northwest. There was an increasing trend during the study period; grassland in summer with better hydrothermal conditions displayed the highest productivity. The effects of various factors on grassland NPP during the study period were further studied. Precipitation was the main influencing factor, followed by solar radiation and NDVI. K value had the least impact on grassland NPP. Annual accumulated temperature (> 0°C) was a negative impact factor, but its correlation was not significant. Our data provide new insight that precipitation is the most important factor affecting the grassland NPP in the fragile and complex ecosystem that is highly vulnerable to climate change. These results provide a reference for revealing the grassland carbon cycle process and its inherent climate-driven mechanism in arid and semi-arid regions, and could also provide basic data for energy conservation during economic development.

## Supporting information

S1 Table The data for interannual changes of grassland NPP. (XLSX)

S2 Table The data for monthly and seasonal variation of grassland NPP. (XLSX)

S3 Table The data for the correlation analysis between annual mean grassland NPP and climate factors. (XLSX)
